# Prevalence, Outcomes and Impact of Disease-Related Complications in the Survival of Multiple Myeloma Patients

**DOI:** 10.3390/hematolrep16010009

**Published:** 2024-02-21

**Authors:** Wachiralak Tothong, Adisak Tantiworawit, Lalita Norasetthada, Chatree Chai-Adisaksopha, Teerachat Punnachet, Nonthakorn Hantrakun, Pokpong Piriyakhuntorn, Thanawat Rattanathammethee, Sasinee Hantrakool, Ekarat Rattarittamrong

**Affiliations:** Department of Internal Medicine, Faculty of Medicine, Chiang Mai University, Chiang Mai 50200, Thailandthanawat.r@cmu.ac.th (T.R.); sasinee.h@cmu.ac.th (S.H.)

**Keywords:** multiple myeloma, hypercalcemia, renal insufficiency, anemia, skeletal-related events

## Abstract

There are limited data regarding the impact of disease-related complications on the survival of multiple myeloma (MM) patients. The primary objective of this study was to determine the prevalence of disease-related complications, including hypercalcemia, renal insufficiency, anemia, and bone lytic lesions in MM patients. The secondary objectives were to determine clinical characteristics, treatment outcomes, and the association of disease-related complications and mortality. A retrospective chart review of MM patients from November 2014 to December 2019 was conducted. A total of 200 MM patients were enrolled. The median age at diagnosis was 63 years. The bone lytic lesion was the most common disease-related complication found in 85% during first-line therapy, followed by anemia (71.5%), renal insufficiency (28.5%), and hypercalcemia (20%). While anemia was the most common complication during the second (51.2%) and third-line therapy (72%). The development of skeletal-related events (SREs) after treatment is a disease-related complication that is associated with decreased overall survival (HR 4.030, 95% CI 1.97–8.24, *p* < 0.001). The most common disease-related complication of MM at initial diagnosis is bone lytic lesions, whereas anemia is more common with subsequent relapses. The presence of SRE after treatment is associated with the increased mortality of MM patients.

## 1. Introduction

Multiple myeloma (MM) is a hematologic cancer characterized by the abnormal proliferation of monoclonal plasma cells that occurs in about 1.8% of all malignancies and 18% of hematologic malignancies [1]. The incidence of disease in Europe is 4.5–6.0 per 100,000 of the population per year [2]. Real-world data from the Swedish Myeloma Registry showed a median age of 71 years, with 28% aged under 65 years. The median overall survival (OS) varied according to age group from 7.8 years in patients aged less than 60 years to 1.5 years for patients aged 80–89 years [3].

The common disease-related complications in MM are “CRAB”, including hypercalcemia, renal insufficiency, anemia, and bone lytic lesions which are also used in the International Myeloma Working Group (IMWG) diagnostic criteria [4]. Renal insufficiency is found in 20–50% of myeloma patients at the first presentation, and about 10% require dialysis [5,6]. The most common cause of kidney injury is cast nephropathy, resulting from the aggregation of toxic monoclonal light chains in renal tubules [5,6]. Osteolytic bone lesions caused by increasing osteoclastic activity are present in 80% of MM patients [7]. Myeloma bone disease increases the risk of skeletal-related events (SREs), including spinal cord compression, pathologic fracture, radiation therapy, or bone surgery [8], and subsequently affects prognosis [9]. Hypercalcemia is one of the complications, which has various clinical presentations, including life-threatening conditions [10]. Anemia in MM is caused by various mechanisms, mainly from myeloma cell-induced erythroblast apoptosis [8]. Previous data from Thailand revealed anemia to be the most common disease-related complication of MM patients [11]. 

The prognosis of MM depends on multiple factors, such as patient characteristics, the stage of disease, cytogenetic abnormalities, and response to therapy [12]. In addition, eligibility for autologous stem cell transplantation (ASCT) and access to novel agents for treatment regimens can improve long-term outcomes [13]. However, data regarding the impact of disease-related complications on survival are still limited. This study aimed to describe the prevalence, outcomes, and impact of disease-related complications (CRAB) across lines of therapy on the survival of MM patients. 

## 2. Material and Methods

We conducted a single-center, retrospective cohort study. Newly diagnosed MM patients, according to the International Myeloma Working Group (IMWG) criteria [4], at Chiang Mai University Hospital from November 2014 to December 2019 were enrolled. This study was approved by the Institutional Review Board of the Faculty of Medicine, Chiang Mai University (Study code: MED-2564-08222). The requirement for consent was waived by the ethics committee.

Data sources were collected from the electronic medical database and medical records. We collected clinical characteristics including age, sex, clinical manifestations, laboratory data including complete blood count, blood chemistry, liver function test, serum protein electrophoresis (SPEP), urine protein electrophoresis (UPEP), serum-free light chain (FLC), serum immunofixation electrophoresis (IFE), lactate dehydrogenase (LDH), beta2-microglobulin, bone lytic lesions (on skeletal radiography, computed tomography [CT], or positron emission tomography [PET]-CT), and tissue biopsy. Staging according to the International Staging System (ISS) [14] was used since cytogenetic risk by conducting fluorescent in situ hybridization (FISH) was not available in all patients. 

Treatment, including the ASCT decision, was based on national guidelines and accessibility to novel agents as well as the ASCT of individual patients. Bisphosphonate (zoledronic acid or pamidronate) was given to all patients during induction therapy unless there was a contraindication. Data regarding treatment, treatment response according to IMWG criteria [15], the treatment outcomes of disease-related complications (CRAB), infectious complications, death, and cause of death at newly diagnosed and subsequent lines of therapy were also gathered. 

### Statistical Analysis 

The primary outcome was to determine the prevalence of the disease-related complications of MM including hypercalcemia (serum calcium > 11 mg/dL), renal insufficiency (serum creatinine > 2 mg/dL), anemia (hemoglobin value < 10 g/dL), bone lytic lesions (one or more osteolytic lesions on skeletal radiography, computed tomography [CT], or positron emission tomography [PET]-CT) as well as SREs (spinal cord compression, pathologic fracture, radiation therapy or surgery to bone) across lines of therapy. The secondary outcomes were to determine the treatment outcomes of the disease-related complications of MM and determine the association between these complications and mortality in MM patients. 

In a previous study [11], the prevalence of disease-related complications of MM was 28.5%, with a confidence interval of 95% and power of 80% with a 10% loss to follow-up rate, a sample size of about 200 patients was calculated. Statistical analyses were performed using Stata 17 (StataCorp, College Station, TX, USA). Descriptive data such as prevalence were presented by number (%), the mean ± SD, and median (interquartile range [IQR] or range) as appropriate. Comparisons of clinical characteristics and laboratory data between cases were performed using Chi-square and Fisher’s exact test for categorical data and Student’s *t*-test or the Mann–Whitney U test for continuous data. Univariable Cox proportional hazard regression models were used to perform the association of disease-related complications and mortality. The multivariable analysis included clinical characteristics, laboratory data, and treatment that had statistically significant results in the univariable analysis. The model was then reduced using a stepwise backward elimination procedure with a statistical significance level of 0.05.

## 3. Results

### 3.1. Clinical Characteristics and Disease-Related Complications

A total of 200 patients with newly diagnosed MM receiving first-line therapy were enrolled. Subsequently, second and third-line therapies were given to 115 (57.5%) and 25 (12.5%) patients, respectively. The median age at diagnosis was 63 years (range 35–92 years), and 111 patients (55.5%) were male. Bone pain was the most common initial clinical manifestation (37%), followed by anemia (25%), renal insufficiency (13%), fracture (11.5%), plasmacytoma (6%), symptoms related to amyloidosis (4.5%), hypercalcemia (1.5%), and fever (1.5%) as shown in Table 1.

Regarding disease-related complications in each line of therapy, bone lytic lesions were the most common complication in newly diagnosed patients receiving their first line of therapy, which was found in 85% of patients, followed by anemia (71.5%), renal insufficiency (28.5%) and hypercalcemia (20%). By contrast, anemia was the most common disease-related complication of patients who underwent second-line therapy (51.2%), followed by bone lytic lesion (35.6%), renal insufficiency (12.1%), and hypercalcemia (5.2%). Likewise, anemia was the most common complication of patients receiving third-line therapy (72%), followed by bone lytic lesions (12%) and renal insufficiency (4%). (Table 2).

For baseline laboratory data, we found that 176 patients (88%) had monoclonal (M) proteins detected from SPEP, whereas 99 patients (49.5%) had M proteins detected from UPEP. Immunoglobulin (Ig) G (44.5%) was the most common subtype, followed by IgA (15%), light chain disease (7.5%), and non-secretory disease (6%). Furthermore, sixteen patients (8%) had plasmacytoma, and ten patients (5%) had biopsy-proven amyloidosis. Of the 200 patients, 80 (40%) suffered from SREs, including 25 (12.5%) with cord compression, 23 (11.5%) with fractures, 20 (10.5%) needing surgery and 26 (13%) undergoing radiation therapy. Regarding staging, 137 patients (68.5%) were classified as Stage III, while 46 patients (23%) and 17 patients (8.5%) were in the ISS II and I, respectively. 

### 3.2. Treatment and Treatment Outcomes

Of the 200 patients, 35 patients (17.5%) underwent ASCT. Novel regimens, including bortezomib, thalidomide, and lenalidomide, were given to 123 patients (61.5%). Response to first-line therapy for at least very good partial response (VGPR) occurred in 43% of patients. 

After treatment, all disease-related complications tended to improve and infrequently resulted in new onset during treatment. Hypercalcemia was entirely improved during the first-line therapy (100%), followed by SRE (96.3%), renal insufficiency (85.9%), and anemia (79.7%). After the second-line therapy, we observed an improvement in SRE (100%), hypercalcemia (94.8%), renal insufficiency (64.3%), and anemia (58.9%). And for the third-line therapy, renal insufficiency experienced 100% improvement, followed by 95% and 85.7% for hypercalcemia and anemia, respectively. (Table 2) There were 9 patients (4.5%) who suffered from SRE after treatment at the median duration from the initial treatment of 5 months (range 2–19 months). Two patients did not receive bisphosphonate, while the seven other patients were administered at the time of SRE. 

Five of the two hundred (2.5%) participants experienced venous thromboembolism (VTE), and all received anticoagulants. Infections were one of the important complications during treatment found at 43.5%, 34.8%, and 36% prevalence during the first, second, and third lines of therapy, respectively, with 36 patient deaths (32.4%) from infections. Pneumonia was the most common infection across lines of therapy (34.5%, 42.5%, and 44.4% during the first, second, and third lines of therapy, respectively).

### 3.3. Overall Survival and Factors Associated with Survival

**Figure 1:**
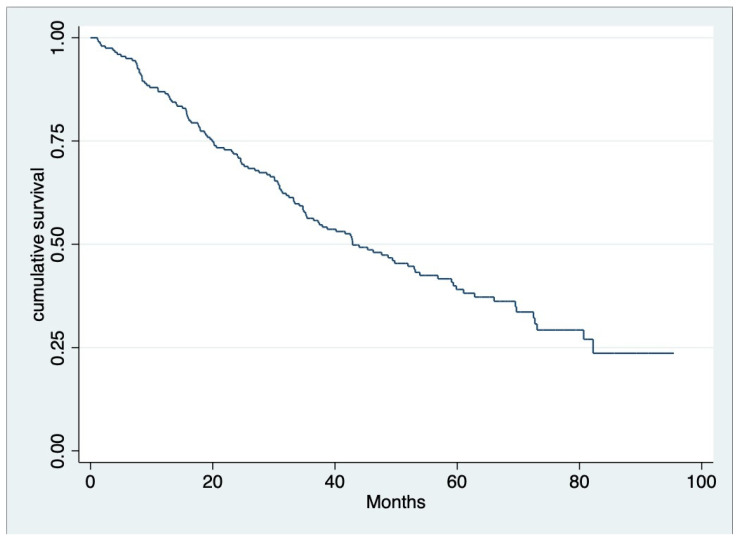
Overall survival of multiple myeloma patients in this study. Median OS was 39.9 months (IQR 19.75, 58.10).

**Figure 2:**
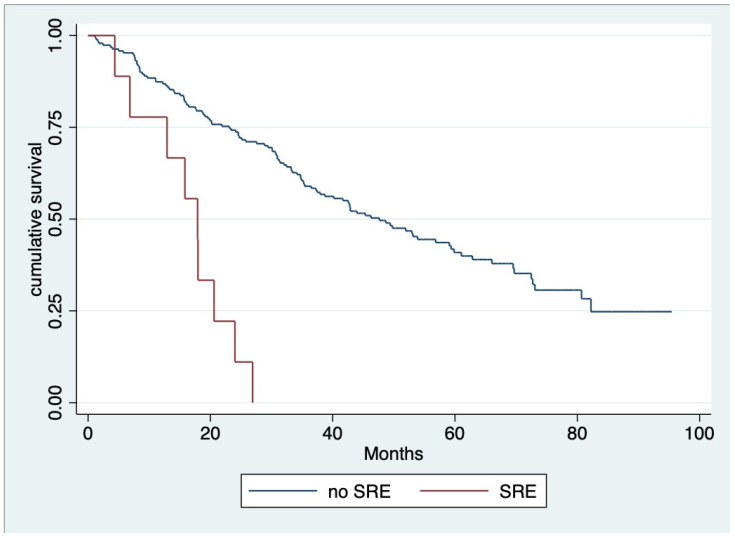
Overall survival regarding skeletal-related event (SREs) after treatment [SRE, N = 9 (4.50%) median OS 17.09 months; no SRE, median OS 47.63 months; and HR 4.030, 95% CI 1.97–8.24, *p* < 0.001].

The median OS of all patients was 39.9 months (IQR 19.8, 58.1) (Figure 1), and throughout the observed period (mean 28.99 ± 19.40 months), 124 out of 200 individuals (62%) died. According to univariate analysis, we found that the age group, serum creatinine, beta-2 microglobulin level at diagnosis, treatment with ASCT, receiving novel agents, response to first-line therapy for at least VGPR, and complication after treatment with anemia or SRE emerged as significant factors for OS. Nevertheless, multivariable analysis revealed that ASCT (hazard ratio [HR] 0.222, 95% CI 0.09–0.51, *p* < 0.001), response to first-line therapy at least for VGPR (HR 0.477, 95% CI 0.32–0.70, *p* < 0.001) and SRE as a complication after treatment (HR 4.030, 95% CI 1.97–8.24, *p*< 0.001) (Figure 2) were factors associated with OS (Table 3).

## 4. Discussion

MM is a hematologic malignancy primarily occurring in the elderly [3]. Our study showed a median age at the onset of disease of 63 years, which is comparable with a previous study in Asia [11]. In addition, ages older than 65 years at the onset of the disease tend to reduce in OS according to univariate analysis. However, the impact of age was not significant regarding multivariable analysis, while ASCT eligibility was a significant factor in increasing OS. This finding supports the importance of ASCT for the management of MM [16] in real-world practice.

Common clinical manifestations of MM were bone pain, anemic symptoms, and renal insufficiency compatible with IMWG criteria for the diagnosis of MM [4]. Bone lytic lesions were the most common disease-related complication, which was found in 85% of patients in first-line therapy. The prevalence was comparable with Japan (80%) and higher than data from Thailand regarding the Asian Myeloma Network study (28.5%) [11]. In addition, SRE was found in 40% of patients, with the proportion of each SRE being comparable and overlapping (cord compression in 12.5%, fractures in 11.5%, surgery for bones in 10.5%, and radiation therapy for bones in 13%). Real-world data from the United States also showed that 41.3% of MM patients had SRE during the 12-month baseline period 60 days on or after MM diagnosis, but the majority of SREs were pathological fractures (27.4%) [7]. Subsequently, 34% of patients experienced SRE during follow-up, which was higher than the results of this study, which found SRE in 6.9% of patients at later lines of therapy. The lower prevalence of disease-related complications such as anemia and SREs during subsequent therapy might be explained by treatment at relapse, which was initiated in patients with significant paraprotein relapse without clinical relapse. Although the proportion was not high, this study revealed the negative impact of SREs after treatment on the survival of MM patients. With a combination of the previous data from a population-based study in Sweden, which found that fracture at diagnosis was a significant risk of death (HR of 1.28) [9], the SRE, both at diagnosis and at follow-up, seemed to be associated with the decreased survival of MM patients. The prevention of SRE by bisphosphonate, such as zoledronic acid, also showed benefits in terms of OS in MM patients [17].

Anemia (71.5%), renal insufficiency (28.5%), and hypercalcemia (20%) had fewer common presentations of MM in this study. Patients with renal insufficiency tended to have lower OS rates in univariate analysis, but this difference was not significant in multivariate analysis. The association of renal failure and survival in MM patients was previously documented, but the reversibility of renal failure was a more important prognostic factor [18]. In this study, the rate of reversibility of renal failure was 86%, which was higher than the study in the pre-novel agent era, which showed that 58% achieved the normalization of serum creatinine [18]. As a result, the impact of renal insufficiency at diagnosis on outcomes in MM is decreased nowadays. Anemia and hypercalcemia are also MM-related complications that have a high rate of response (100% and 80%, respectively) after treatment, and the impact of both complications is not demonstrated in this study. However, a previous systematic review showed anemia to be associated with shorter survival times in many types of cancer, including MM [19].

Patients with MM are at high risk of VTE, with an estimated incidence of more than 10% during the disease [20]. However, only 2.5% of patients developed VTE during this study, which supports the Asian race as the protective factor of VTE in some risk assessment models [21]. Infection is also a common complication of MM patients, which might be related to disease as well as treatment. In this study, it occurred at approximately 30–40% across lines of therapy and led to mortality in about one-third of MM patients. The population-based study from Sweden revealed a slightly lower proportion of MM patients who died from infection (22%) but still demonstrated a seven-fold risk of infection compared to the normal population [22]. The higher rate of infection-related mortality in this study might partially explain the shorter median OS of MM patients at 39.9 months compared to other studies [3,11]. In addition, it might result from the limited accessibility of novel agents (61.5%) and ASCT (17.5%).

There are some limitations to this study. First, due to the nature of a retrospective study, some data were missing, and bias may have occurred. Second, data regarding the cytogenetic study determined by FISH and minimal residual disease testing were not available in the majority of patients. And lastly, due to a single-center study with a limited number of patients, these findings should be validated in further studies. Nevertheless, this study provides additional data about the impact of SREs after treatment on the survival of MM patients.

## 5. Conclusions

The most common disease-related complication of MM at initial diagnosis was bone lytic lesions, whereas anemia was more common in subsequent relapses. The presence of SREs after treatment is associated with increased mortality.

## Figures and Tables

**Table 1:** Clinical characteristics of patients.

	Total (N = 200)
Age of onset (years)	63.07 ± 10.82
Sex: male	111 (55.50%)
Duration from onset (years)	5.08 ± 1.43
3	33 (16.50%)
4	48 (24%)
5	33 (16.50%)
6	45 (22.50%)
7	37 (18.50%)
8	4 (2.00%)
Clinical manifestations	
Bone pain	74 (37.00%)
Anemic symptom	48 (25.00%)
Symptom related to renal failure	26 (13.00%)
Fracture	23 (11.50%)
Plasmacytoma	12 (6.00%)
Symptom related to amyloidosis	9 (4.50%)
Symptom related to hypercalcemia	3 (1.50%)
Fever	3 (1.50%)
Others	15 (7.50%)

**Table 2:** Prevalence and outcomes of disease-related complications of multiple myeloma across lines of therapy.

	First Line (N = 200)	Second Line (N = 115)	Third Line (N = 25)
Ca (mean ± SD, mg/dL)	9.82 ± 1.81	9.27 ± 1.19	9.12 ± 0.52
>11	40 (20.00%)	6 (5.22%)	0
Improved	40 (100%)	6 (100%)	0
Recurrent	0	0	0
Cr (mean ± SD, mg/dL)	2.08 ± 2.33	1.42 ± 1.13	1.28 ± 0.98
>2	57 (28.50%)	14 (12.17%)	1 (4.00%)
Improved	49 (85.96%)	9 (64.29%)	1 (100%)
Recurrent	2 (1.40%)	11 (10.89%)	2 (8.33%)
Hb (mean ± SD, g/dL)	8.97 ± 2.21	10.20 ± 2.25	10.74 ± 1.89
≤10	143 (71.50%)	59 (51.20%)	18 (72.00%)
Improved	114 (79.72%)	33 (55.93%)	6 (33.33%)
Recurrent	3 (5.26%)	14 (23.73%)	2 (11.11%)
Bone lytic lesion	170 (85.00%)	41 (35.65%)	3 (12.00%)
SRE	80 (40.00%)	8 (6.96%)	0
Cord compression	24 (12.50%)	4 (3.48%)	0
Surgery	21 (10.50%)	2 (1.74%)	0
RT	26 (13%)	9 (7.83%)	0
Improved SRE	77 (96.25%)	8 (100%)	0
Recurrent SRE	6 (5%)	5 (4.67%)	1 (4%)

Ca: calcium; Cr: creatinine; Hb: hemoglobin; SREs: skeletal-related events; RT: radiation therapy.

**Table 3:** Multivariable analysis for overall survival.

	HR	95% CI	*p* Value
Age ≥ 65 years	1.200	0.78–1.82	0.395
Cr > 2 mg/dL	1.133	0.72–1.77	0.582
Beta-2 microglobulin ≥ 3.5 mg/L	1.405	0.77–2.54	0.262
Underwent ASCT	0.222	0.09–0.51	*p* < 0.001
Received novel agents	1.011	0.65–1.56	0.961
Response to first-line therapy ≥ VGPR	0.477	0.32–0.70	*p* < 0.001
Complication after treatment: anemia	1.307	0.77–2.21	0.319
Complication after treatment: SRE	4.030	1.97–8.24	*p* < 0.001

Cr: creatinine; ASCT: autologous stem cell transplantation; VGPR: very good partial response; SRE: skeletal-related event.

## Data Availability

The data that support the findings of this study are available from the corresponding author, [ER], upon reasonable request.
